# The P66 time-resolved VUV spectroscopy beamline at PETRA III storage ring of DESY

**DOI:** 10.1107/S1600577525007568

**Published:** 2025-09-17

**Authors:** Yevheniia Smortsova, Oksana Chukova, Marco Kirm, Vitali Nagirnyi, Vladimir Pankratov, Aleksandr Kataev, Aleksei Kotlov

**Affiliations:** a Deutsches Elektronen-Synchrotron DESY Notkestr. 85 22607Hamburg Germany; b Institute of Physics University of Tartu W. Ostwald Str. 1 50411Tartu Estonia; c Institute of Solid State Physics University of Latvia Kengaraga 8 RigaLV-1063 Latvia; RIKEN SPring-8 Center, Japan

**Keywords:** luminescence, vacuum ultraviolet excitation, time-resolved spectroscopy, reflectance spectra

## Abstract

The paper presents a comprehensive description of the construction features of the P66 beamline at the PETRA III storage ring as well as the experimental possibilities offered by this unique facility to the research community. The beamline is dedicated to time-resolved photoluminescence spectroscopy experiments that require high flux, high energy resolution and pulsed excitation at ultrahigh vacuum conditions. The excitation photon energies available at the beamline cover the range from 3.7 to 40 eV with two Al- and Pt-coated diffraction gratings in normal incidence geometry. Two secondary monochromators equipped with several detectors are used to register the photoluminescence signal in the spectral range from 115 to 1200 nm. The pulsed nature of the synchrotron radiation at PETRA III allows the accumulation of luminescence decay kinetics within a time interval of 192 ns (timing mode) or 16 ns (brightness mode) with a time resolution of 130 ps. Time-resolved luminescence spectroscopy studies are performed using the method of time-correlated single photon counting. The paper also reviews recent advances, as well as the most common and important scientific cases of experimental research conducted at the beamline. Plans for the future development of the beamline, along with the expansion of experimental capabilities and the implementation of additional applications of the facility, are discussed.

## Abstract 2

A comprehensive description of the technical details and science case of the recently opened beamline for time-resolved VUV spectroscopy at DESY, PETRA III, is presented.

## 1. Introduction

The application of synchrotron radiation light sources for research in materials science has experienced rapid development over the last decades. These facilities have revolutionized materials research by offering unique capabilities for studying the structure, electronic properties and energy relaxation dynamics of materials at unprecedented levels of detail. The advantages of applying synchrotron radiation in materials science are its high brightness, broad spectral range, narrow angular collimation, high degree of polarization and pulsed time structure that enable precise investigation of materials (Mobilio *et al.*, 2014 ▸). In particular, the application of pulsed synchrotron radiation is the most efficient way to provide necessary time and energy resolution of the excitation in the vacuum ultraviolet (VUV) photon energy range. The luminescence arising from such excitation is used to study the properties of both intrinsic and extrinsic impurity/defect states, energy transfer processes, to determine the bandgap values of materials, to elucidate energy relaxation pathways and recombination mechanisms of electronic excitations, and to study surface structure (Omelkov *et al.*, 2022 ▸; Kamenskikh *et al.*, 2020 ▸; Pankratov *et al.*, 2022 ▸). Such experiments are extremely important for investigating the electronic structure of wide bandgap materials because of the lack of alternative laboratory radiation sources. Indeed, most conventional optical materials (like glass, quartz or standard mirror coatings) absorb strongly at these wavelengths, making it difficult to build optical systems with tuneable monochromators, lenses or even windows. Atmospheric gases also absorb strongly below 190 nm, so experiments require vacuum environments. Additionally, generating tuneable VUV light typically involves complex and inefficient nonlinear processes such as high harmonic generation, which demand ultrafast lasers and precise control. As a result, synchrotron radiation remains the primary source of intense and tuneable VUV light, far exceeding what is feasible in typical laboratory settings. The P66 beamline offers a uniquely powerful combination of tuneability and pulsed operation, providing intense, broadband radiation that can be precisely selected across the VUV range required for advanced VUV spectroscopy and time-resolved measurements.

In this paper, we present a description of the beamline setup and the available experimental techniques at the P66 beamline located at the PETRA III synchrotron storage ring, DESY (Hamburg, Germany) (Franz *et al.*, 2006 ▸; Drube *et al.*, 2016 ▸). The new P66 beamline is a successor to the well known SUPERLUMI station at the DORIS III storage ring (Zimmerer, 2007 ▸; Möller *et al.*, 1986 ▸) and is specially designed for the time-resolved photoluminescence (TRPL) experiments on solids excited by VUV photons. The first commissioning experiments at the beamline took place at the end of 2021 and it was reported as the first dipole beamline at PETRA III at the 13th International Particle Accelerator Conference (Wanzenberg *et al.*, 2022 ▸). Here we describe the optics and instrumentation installed at the beamline, discuss several measurement techniques implemented, and present some test and new experimental results.

## 2. The PETRA III P66 beamline experimental setup

**Figure 1:**
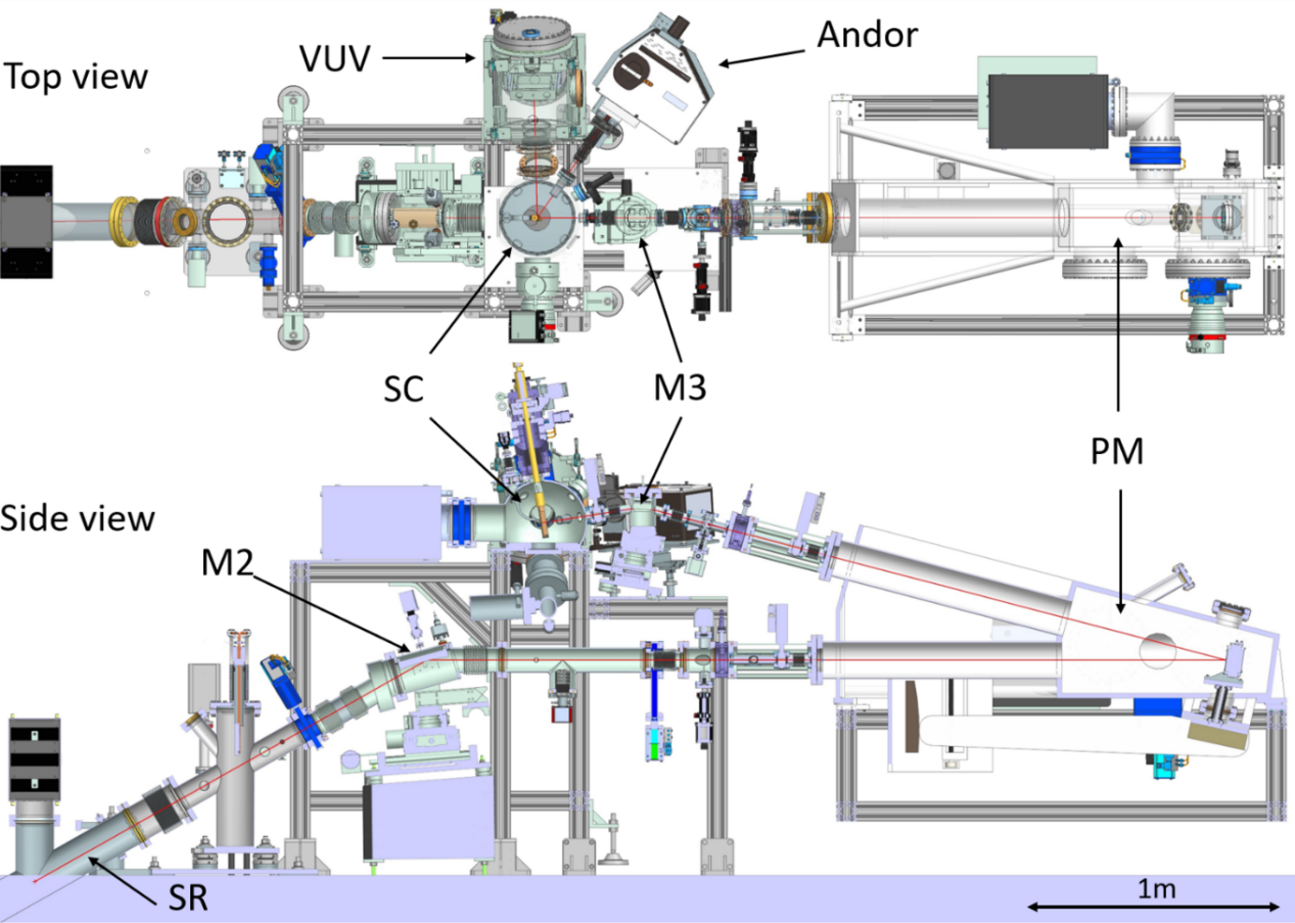
The PETRA III P66 beamline setup layouts (top and sectional side view). SR: synchrotron radiation; M2, M3: focusing mirrors; PM: primary monochromator; secondary VUV and Andor monochromators; SC: sample chamber of P66 beamline.

The P66 experimental hutch is situated on top of the radiation shielding of PETRA III. To obtain a high flux at the entrance slit of the primary monochromator (PM), a 2.3 m-long bending magnet (BM) with a 191.73 m radius and a magnetic field of 0.10439 T is used as a source. On these 2.3 m, the electron beam is bent by 12 mrad. The two optical mirrors, M1 and M2, were installed in order to collect radiation and focus the photon beam in the horizontal and vertical planes on the entrance slit of the PM. The optical layout top and side views are shown in Fig. 1 ▸. The mirror M1 is located 10.8 m from the source and placed in the PETRA III synchrotron tunnel in the plane of the electron beam in storage ring. M1 covers 12 mrad of the horizontal acceptance and reflects the radiation at the angle of 30° upwards to the experimental hutch. The M2 mirror is located 21 m from the source, inside the experimental hutch, and accepts 2 mrad of the synchrotron radiation beam in the vertical plane. The specifications of the optical components of the P66 beamline, various timing and spatial parameters as well as spectroscopy equipment are given in Table 1 ▸.

The 2 m McPherson normal incidence PM has a grating holder for two *in situ* interchangeable gratings (Al- and Pt-coated, 1200 lines mm^−1^), which are operated alternately. The monochromated incident beam is focused by a toroidal mirror M3 onto a sample holder inside the sample chamber. An He/N_2_-flow cryostat with a high-temperature option or a cost efficient closed-cycle He-cryostat can be used for sample cooling under ultra-high vacuum (UHV) conditions. The luminescence can be analysed by two monochromators. The monochromator for the UV–Vis–near-infrared range is a 0.3 m Kymera 328i (Andor) device with an F/4.1 aperture (200–1200 nm) and with a nominal dispersion of ∼10 nm mm^−1^. It is equipped with three interchangeable 300 lines mm^−1^ gratings blazed at 300, 500 and 1200 nm optimized for different spectral regions. The luminescence from the sample is collected and focused on the Kymera monochromator entrance slit with two quartz lenses. The Kymera monochromator has two output ports, the imaging one is equipped with a CCD camera (Newton 920) and the classical one with a set of interchangeable photomultiplier tube (PMT) detectors: Hamamatsu R6358 and R3809U-50. A 12-position filter wheel from Thorlabs allows the selection of an appropriate longpass filter with a cut-on wavelength from 280 to 715 nm. The empty slot of the filter wheel is reserved for the measurements of luminescence in the UV range, between 200 and 300 nm.

**Figure 2:**
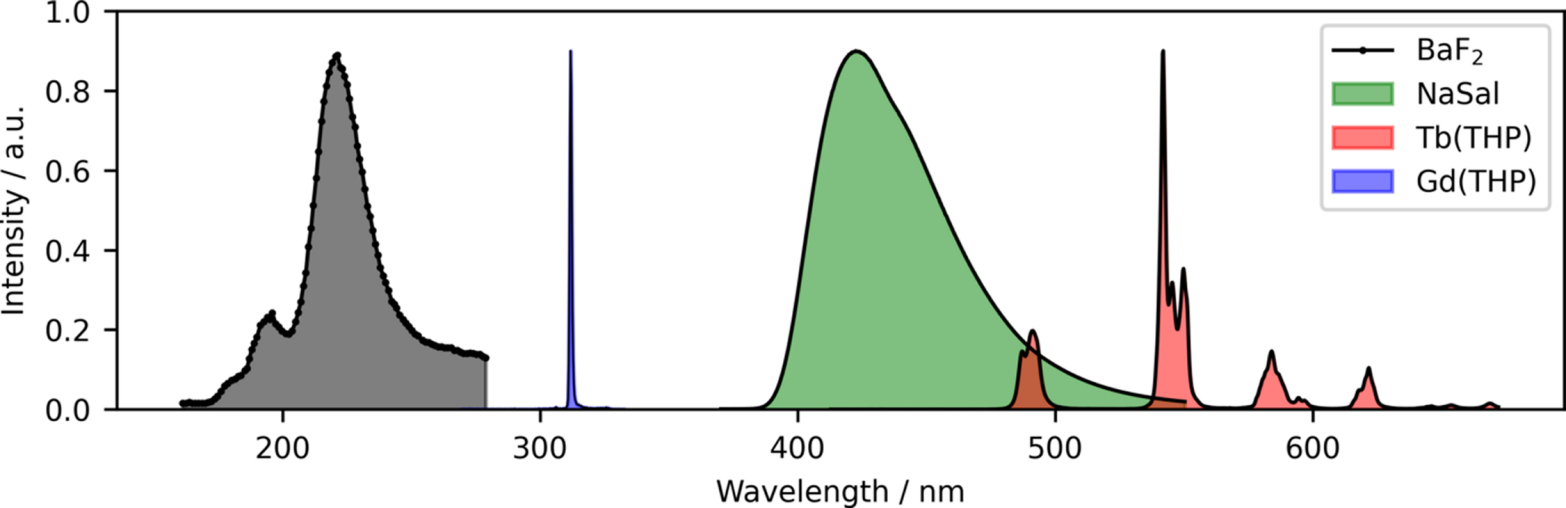
Emission spectra recorded with secondary VUV and UV–Vis Andor monochromators. Black: BaF_2_ cross luminescence, λ_exc_ = 56 nm, VUV Solar blind PMT detector, 1 mm exit slit. Blue and red: emission of Gd(THP)Cl_3_ and Tb(THP)Cl_3_ complexes, respectively, λ_exc_ = 160 nm, Andor monochromator and CCD Newton camera. Green: luminescence of sodium salicylate, λ_exc_ = 160 nm.

The monochromator for the VUV range is a specially designed device (Pouey mounting: F/2.8) without an entrance slit and with variable density of lines of the diffraction grating (Gürtler *et al.*, 1983 ▸). Its operating range is from 115 to 320 nm, currently limited by the solar-blind PMT (Hamamatsu R6836), but there is the second output for the windowless MCP detector sensitized by a CsI coating. The spectral resolution of the VUV monochromator depends on the opening of the exit slit and the size of the excitation beam on the sample. The best resolution achieved so far is 0.9 nm. The upgrade of the detectors is planned, in particular the implementation of a new windowless MCP detector for shorter wavelengths. The combination of the two aforementioned secondary monochromators provides great flexibility over a wide range of wavelengths for the PL analysis as shown in Fig. 2 ▸.

There is a specially designed port for reflectance measurements from the crystals under 15° angle of incidence with respect to the excitation beam. The reflectance spectra can be recorded simultaneously with the excitation spectra, which increases efficiency of data collection. The reflected VUV radiation is converted to blue emission with the help of a sodium salicylate layer on the inner side of the output port. Outside the vacuum chamber, there is a filter unit mounted with a set of grey filters (0.1–30% transmission) allowing the adjustment of the reflection signal level depending on the surface quality (cleaved, polished, as grown) of the crystals. The blue emission is detected with a Hamamatsu R1166 PMT through a bandpass filter selecting sodium salicylate luminescence with the maximum at 420 nm.

The sample environment is designed to achieve UHV conditions down to 1.0 × 10^−10^ mbar, necessary to avoid the absorption of the VUV radiation by air and to keep the sample surface free of contamination during sample cooling. Indeed, high absorption coefficients at the edge of the material fundamental absorption (∼10^6^ cm^−1^) result in the penetration depth of the VUV radiation on the order of ∼10 nm and any surface contamination can have a serious impact on the experimental results. The beamline provides two KONTI flow-type cryostats from the CryoVac company for cooling samples down to liquid nitro­gen/helium temperatures or heating samples up to 700 K. For one of the flow-type cryostats, a device for the crystal cleavage under the UHV conditions is in commissioning. A closed-cycle cryostat (ARS CS204SB) can also be installed to reduce the operating costs for liquid helium experiments and can be operated in the temperature range 10–350 K for most experiments. All above-mentioned cryostats are specially designed for the UHV conditions of the P66 beamline. Because of the UHV, the beamline accepts only solid samples: crystals, powders, glasses, ceramics, *etc*. Up to 40 samples of a few millimetres in size can be glued on a double-sided copper sample holder (100 mm × 22 mm) with a silver conductive paint to obtain a good heat conductivity for reaching low temperatures. The temperature of the flow-type and closed-cycle cryostats during their operation is regulated and monitored by Cryovac TIC 500 and Lake Shore 335 controllers. A calibrated Si diode (DT-670-SD-1.4H, 12 mK accuracy) sensor and a Pt sensor from Lake Shore are installed and used to monitor the temperature of the sample holder. The Si diode sensor is affixed to the copper sample holder near the sample position, providing an estimate of the sample temperature. The achievable temperature range and stabilization time depend on both the target temperature and the sample material, and can be monitored through the stability of the luminescence signal.

## 3. Luminescence excitation spectroscopy

**Figure 3:**
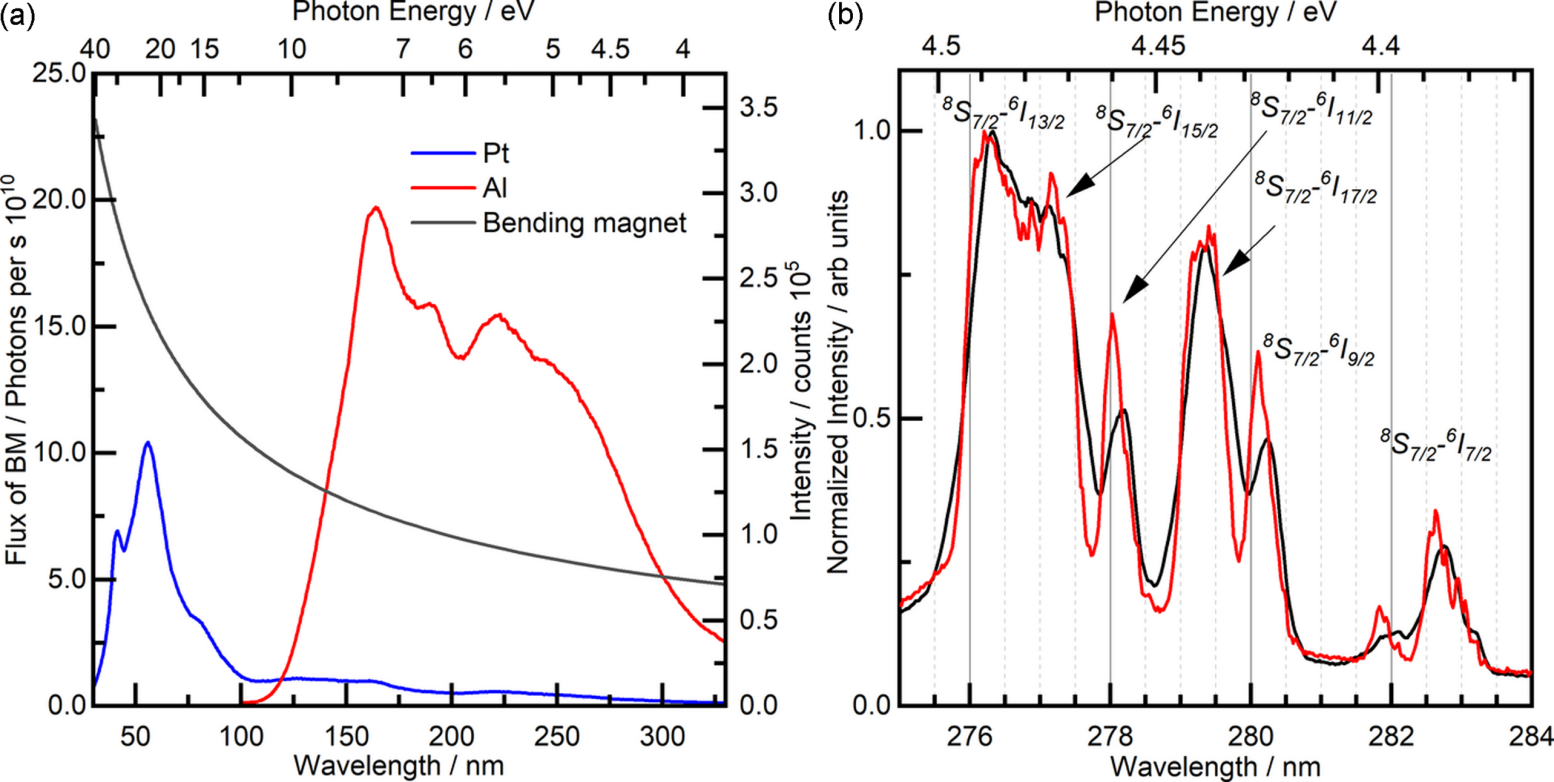
(*a*) Excitation spectra of a sodium salicylate reference sample using the Al (red curve) and the Pt (blue curve) gratings. The black curve represents the calculated flux from the BM. (*b*) Excitation spectra of Gd_3_Ga_5_O_12_ in the spectral range of the Gd^3+^^8^*S*_7/2_–^6^*I*_*J*_ transitions, while monitoring the ^6^*P*_*J*_–^8^*S*_7/2_ emission (317 nm) of Gd^3+^ at 10 K, recorded with a standard exit slit width of 2.5 mm (black curve) and the smallest controllable exit slit 0.1 mm (red curve) of the PM.

The PETRA III P66 experimental possibilities allow us to record luminescence excitation and reflectance spectra under excitation by the 3.7–40 eV photons. The excitation spectra of the reference sodium salicylate sample are presented in Fig. 3 ▸(*a*) together with the calculated photon flux of the BM. The flux spectra were recorded using both Al and Pt gratings of the PM. The sodium salicylate sample was taken as a reference material because it has constant quantum yield (around 60%) in the 30–360 nm excitation range (Kristianpoller, 1964 ▸; Samson & Ballard, 1968 ▸; Iglesias *et al.*, 2017 ▸; Adams *et al.*, 1981 ▸) and has been used over the history of luminescence spectroscopy under VUV excitation. Given the luminescence intensity of sodium salicylate does not depend on the excitation energy, the curves shown in Fig. 3 ▸(*a*) directly exhibit total spectral efficiency of the optics of the setup and the gratings of the PM. The excitation spectra of sodium salicylate emission with a maximum at 420 nm are further used for the normalization of the excitation and the reflectance spectra recorded from the sample under study.

The width of the entrance slit of the PM can be continuously adjusted manually, up to the maximum value of 2.5 mm, to operate either with high-resolution or in high-flux mode. Fig. 3 ▸(*b*) shows low-temperature high-resolution excitation spectra of Gd^3+^ emission (^6^*P*_*J*_–^8^*S*_7/2_) recorded with different slit widths of the PM in the range of the ^8^*S*_7/2_–^6^*I*_*J*_ 4*f* transitions. The lines were attributed according to the references (Komar *et al.*, 2016 ▸; Wegh *et al.*, 1997 ▸). All electronic transitions can be successfully resolved even when the slit of the PM is fully open [Fig. 3 ▸(*b*), black line] due to high linear dispersion of the PM. The fine vibrational structure shown as narrow lines [down to 0.1 nm full width at half-maximum (FWHM)] was resolved upon decreasing the slit to 0.1 mm.

## 4. Time-resolved experiments

**Figure 4:**
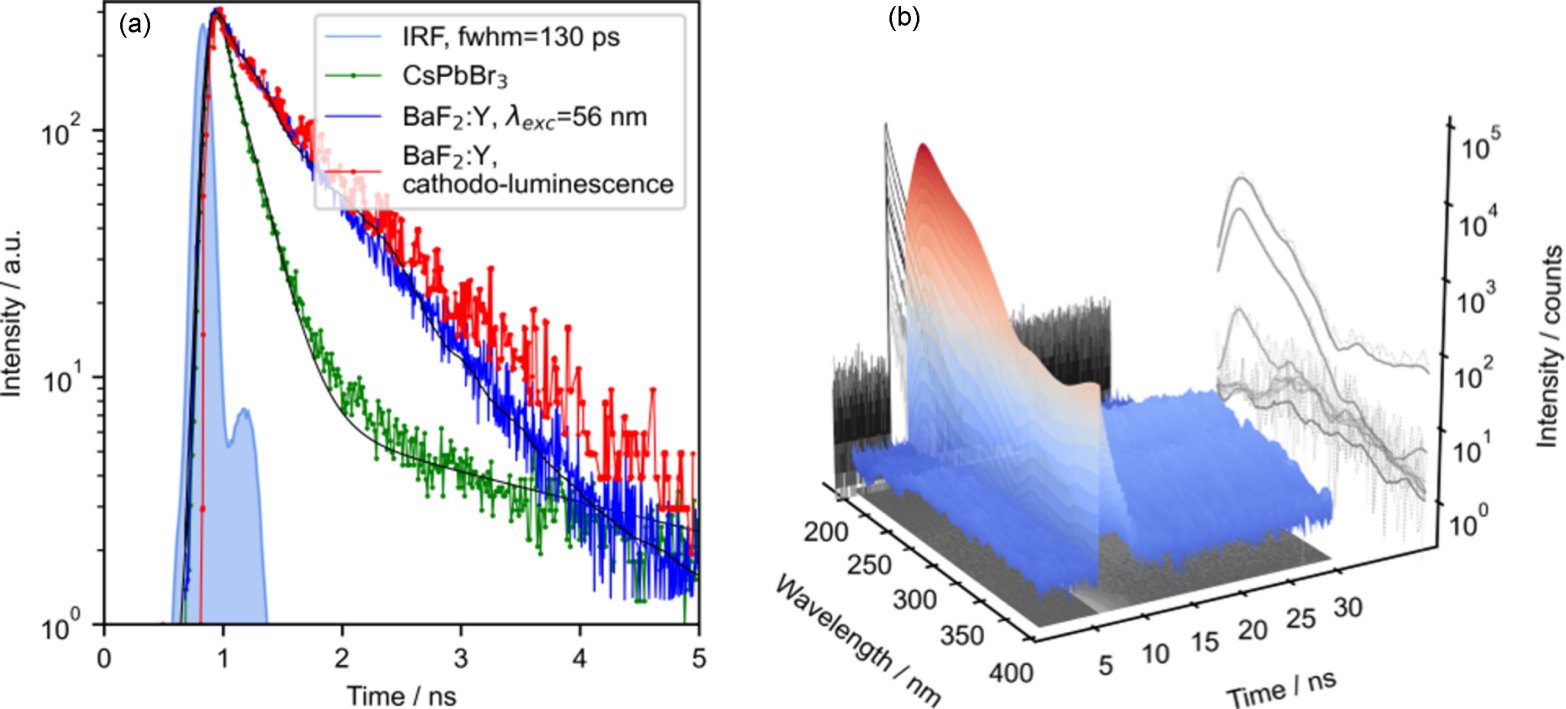
(*a*) Luminescence decay kinetics for 225 nm emission of BaF_2_:Y, excited by a λ_exc_ = 22.8 eV (56 nm) synchrotron pulse (blue curve) and by pulsed CL of BaF_2_:Y (red curve); luminescence decay kinetics of 520 nm emission in CsPbBr_3_ nanocrystals under VUV excitations (green curve) and the IRF of the MCP-PMT detector (light-blue curve with shaded area). The iterative reconvolution biexponential fit results are presented as black curves for CsPbBr_3_ and BaF_2_:Y, excited by the synchrotron pulse. (*b*) Time-resolved emission spectra of a BaF_2_ crystal excited by 22.14 eV photons at 295 K.

TRPL measurements at the PETRA III P66 beamline are possible due to an advantageous combination of the pulsed nature of synchrotron radiation, fast detectors and high-speed electronics. Photoluminescence (PL) decay kinetics are measured using a modified time-correlated single-photon counting (TCSPC) technique (Saaring *et al.*, 2021 ▸). In the 40-bunch synchrotron mode (timing mode) the interval between the sequent bunches is 192 ns, which provides a typical for TCSPC time window to study a number of processes related to the relaxation of the excited states. In the multi-bunch mode, the bunch separation is about 16 ns, which restricts the available window but it still allows the study of fast components in PL decay kinetics. In order to accumulate, process and analyse incoming pulses, the setup of P66 exploits a high-resolution time-to-digital converter Chronologic xTDC4-PCIe, synchronized with the PETRA III bunch clock module (Klute *et al.*, 2011 ▸). The card is triggered by a bunch clock signal and returns the time stamps of the luminescence photons detected by the PMT/MCP-PMT, amplified and processed with a constant fraction discriminator. The recorded timestamps of the signals are used to build a histogram, shifted and wrapped in order to construct a single decay curve proceeding from the storage ring bunch structure by the software developed at the beamline. The Cronologic xTDC4 time-to-digital converter has a 13.0208333 ps bin width and records timestamps within the 7.685 µs time interval (the full revolution of an electron bunch in the PETRA III storage ring). Depending on the configuration used, the temporal resolution of the instrument was measured to be 130 ps using the Hamamatsu R3809U-50 MCP-PMT [Fig. 4 ▸(*a*)], 800 ps for Hamamatsu R6358 PMT and 2 ns for Hamamatsu Solar Blind R6836 PMT. The total time resolution of the setup [evaluated using the instrument response function (IRF)] is determined by the duration of a synchrotron pulse [related to the electron bunch duration, 103 ps (Göries *et al.*, 2016 ▸)], time response of the detector (45 ps IRF FWHM of Hamamatsu R3809U-50 MCP-PMT), signal transport rate and the parameters of signal processing electronics. The contribution of the latter two components was estimated from the total 130 ps FWHM using the relation
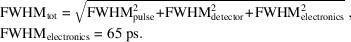
Fast emission decay kinetics of the BaF_2_:Y single crystal and CsPbBr_3_ perovskite nanocrystals are exemplified in Fig. 4 ▸(*a*). The decay kinetics of BaF_2_:Y, recorded at P66 under 22.8 eV excitation agrees in general with the data obtained using pulsed cathodoluminescence (CL) (100 keV), showing the main CL decay component of 686 ps with a contribution of a fast 110 ps component. The CL decay also contains a well distinguished faster component of about 240 ps with a comparable contribution to the total light sum, emerging due to excitation density effects and surface quenching of CL. Similarly, the 520 nm emission decay of CsPbBr_3_ nanoparticles is shown to be dominated by a 186 ps component, accompanied with a longer component of 3.35 ns.

**Figure 5:**
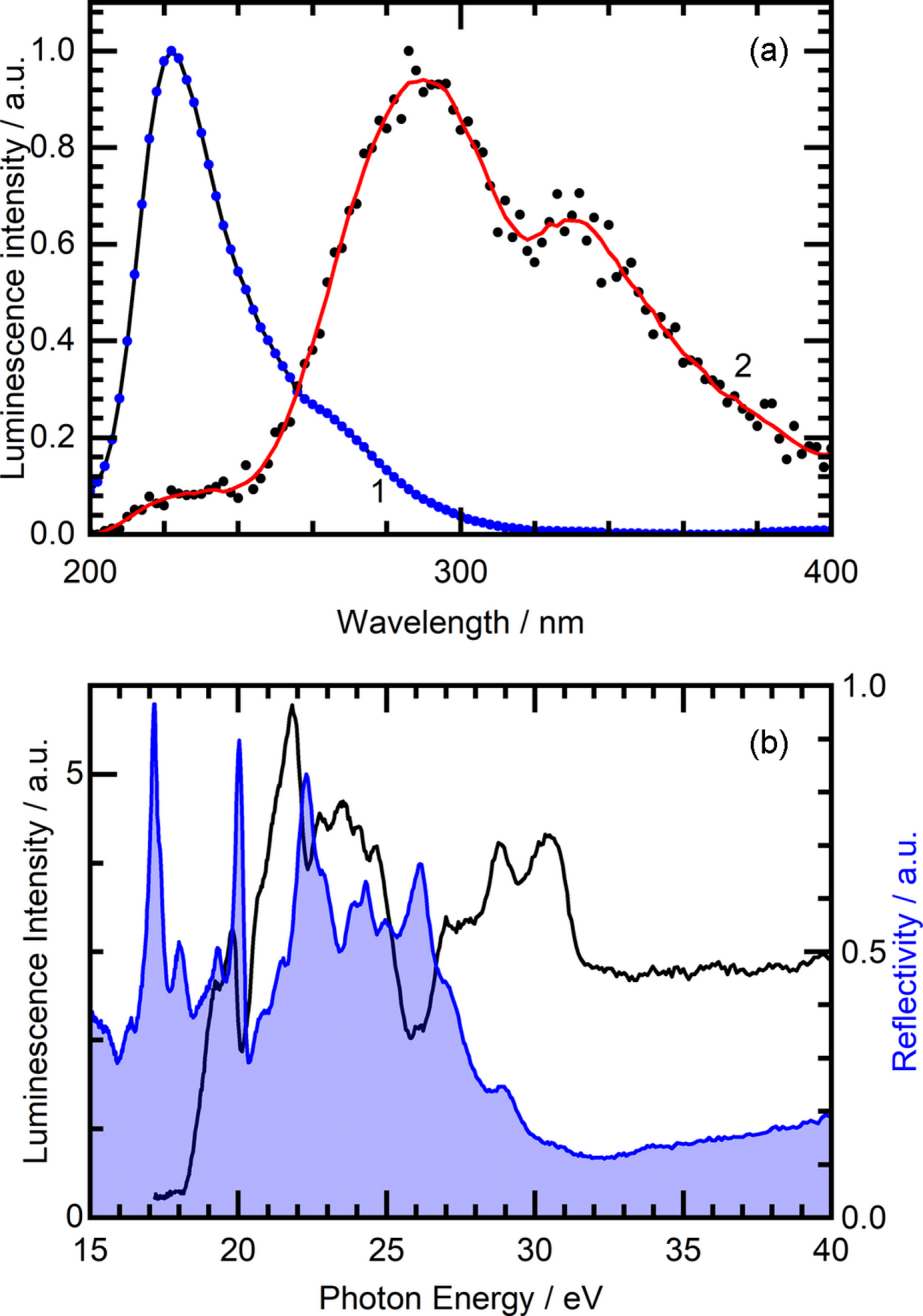
(*a*) Normalized time-resolved emission spectra of a BaF_2_ crystal excited by 22.14 eV photons at 295 K, integrated in the time window of Δ*t* = 400 ps with δ*t* = 0 ns delay with respect to the excitation pulse (1) and in the time window of Δ*t* = 160 ns with δ*t* = 10 ns delay (2). (*b*) Reflectance (blue line with shaded area) and excitation (black line) spectra for the 225 nm cross-luminescence of the BaF_2_ crystal at 15 K.

The P66 experimental hardware configuration with a specially developed data acquisition software allows us to record time-resolved emission and excitation spectra using both the timing and the intensity modes of PETRA III. Fig. 4 ▸(*b*) demonstrates the time-resolved emission spectra of a BaF_2_ crystal excited by 22.14 eV photons at 295 K. This is a result of recording the intensity decays for each emission wavelength in the 200–400 nm interval with a step of 2 nm. Such 3D datasets (decay curves at all wavelengths) are stored in text format and can be conveniently processed after experiments using user developed software tools as well as commercial software for data analysis. For example, the data processing and plotting shown in Figs. 4 ▸(*a*) and 4 ▸(*b*) were performed using a Python 3 script employing the *SciPy* and *Matplotlib* libraries. Two emission spectra presented in Fig. 5 ▸(*a*) show the result of luminescence signal integration in time windows of different length. Spectrum 1 was integrated within the time interval 0–400 ps, and therefore depicts well fast emission components, like the cross-luminescence at 225 nm (Kirm *et al.*, 2001 ▸), but also scattered synchrotron radiation. Spectrum 2 results from the integration in the time interval 10–170 ns, and therefore the self-trapped excitons (STE) emission at 290 nm (Kirm *et al.*, 2001 ▸) with near microsecond lifetime becomes more pronounced. As expected, the fast emission practically does not contribute to this spectrum, and only the STE emission is ready for further analysis. This is a practical way to discriminate contributions with different kinetic constants.

Fig. 5 ▸(*b*) depicts a reflectance spectrum and time-integrated excitation spectrum of cross-luminescence recorded for the 225 nm band at low temperatures (15 K). The excitation onset at 18.2 eV corresponds to the photoionization of the Ba 5*p* core level. The cross-luminescence is the radiative transition of this formed hole to the valence F 2*p* band. The quality of reflectance and excitation spectra demonstrates the expected performance of the P66 beamline in deep VUV using the Pt-coated grating. The observed spectral features are in good agreement with the earlier investigations of BaF_2_ crystals at the SUPERLUMI station (Kirm *et al.*, 2001 ▸).

## 5. Thermally stimulated luminescence

**Figure 6:**
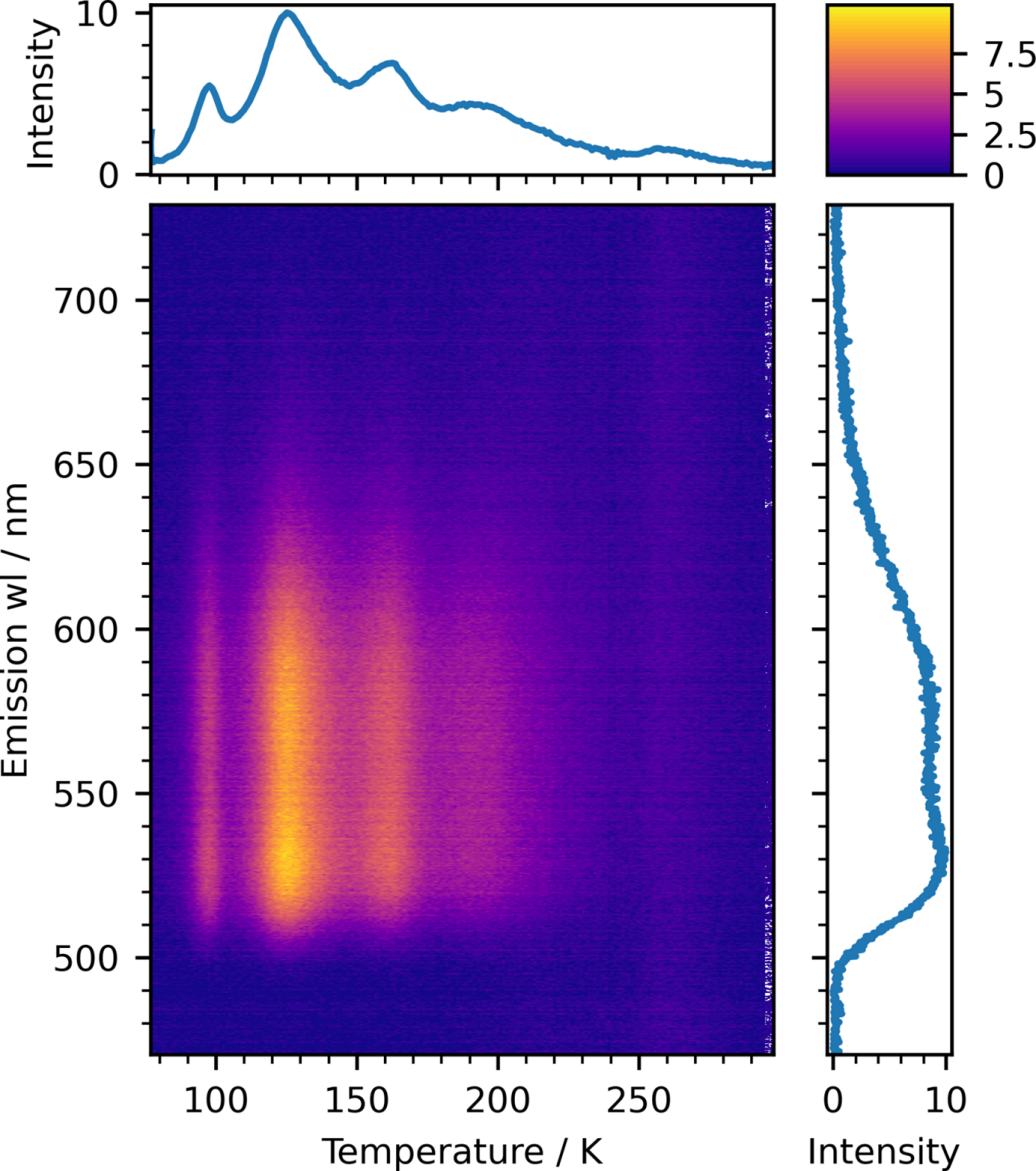
TSL spectra of the eutectic garnet Al_2_O_3_–Y_3_Al_5_O_12_:Ce, recorded after exposure to 7.75 eV radiation for 30 min. The heating rate was 5 K min^−1^. The top panel depicts a classical glow curve: an integrated luminescence intensity as a function of temperature. The right panel shows the integrated over temperature spectral composition of the TSL signal.

Thermally stimulated luminescence (TSL) can be recorded using both PMT and CCD detectors, after exposing the sample at low temperatures (*e.g.* 10 K) to the radiation within the UV–VUV energy range 3.7–40 eV. TSL spectra of an eutectic garnet Al_2_O_3_–Y_3_Al_5_O_12_:Ce were recorded after irradiation by 7.75 eV (160 nm) light for 30 min and then heating the sample with a heating rate of 5 K min^−1^. The heating rate 5 K min^−1^ was chosen as a compromise between measurement resolution and experimental stability, ensuring reliable temperature control and minimizing thermal lag across the sample. The maximum achievable heating rate of the system, while maintaining uniform temperature and stable operation, has not been estimated yet. The spectra were recorded every 10 s. The resulting 3D map is shown in Fig. 6 ▸, as well as the integrated intensity over the full spectral range versus temperature (top panel, so-called ‘glow curve’) and the integrated TSL spectrum over the full temperature range (right panel). The possibility to selectively pump different electronic states, choosing the excitation energy with the PM, is a significant advantage of the TSL at P66 compared with the conventional technique using the γ- or X-ray exposure. On the other hand, the doses achievable are not very high in comparison with classical TSL spectroscopy.

## 6. Science case

In this section, the scientific background of the beamline is described, focusing on the papers that involve the results obtained at the PETRA III P66 beamline and published since its opening in September 2021.

### 6.1. Physical phenomena that occur under VUV radiation in wide-bandgap materials

The VUV energy range accessible at P66 presents particular advantages for the study of electronic structure and excited state dynamics of wide-bandgap materials. Band-to-band transitions and near-edge host absorption processes, in particular excitonic properties, cannot be sufficiently investigated at conventional laboratory facilities because of the lack of suitable light sources needed for the corresponding excitation energies. In addition, the PETRA III P66 beamline allows us to obtain information on impurity states of wide-bandgap materials doped with rare earth ions that act as emission centres. This can be clearly seen from the rich excitation and emission spectra, obtained at P66 [*cf*. literature (Zdeb-Stańczykowska *et al.*, 2025 ▸; Majewski-Napierkowski *et al.*, 2024 ▸), and Figs. 2 ▸ and 3 ▸(*b*)].

**Figure 7:**
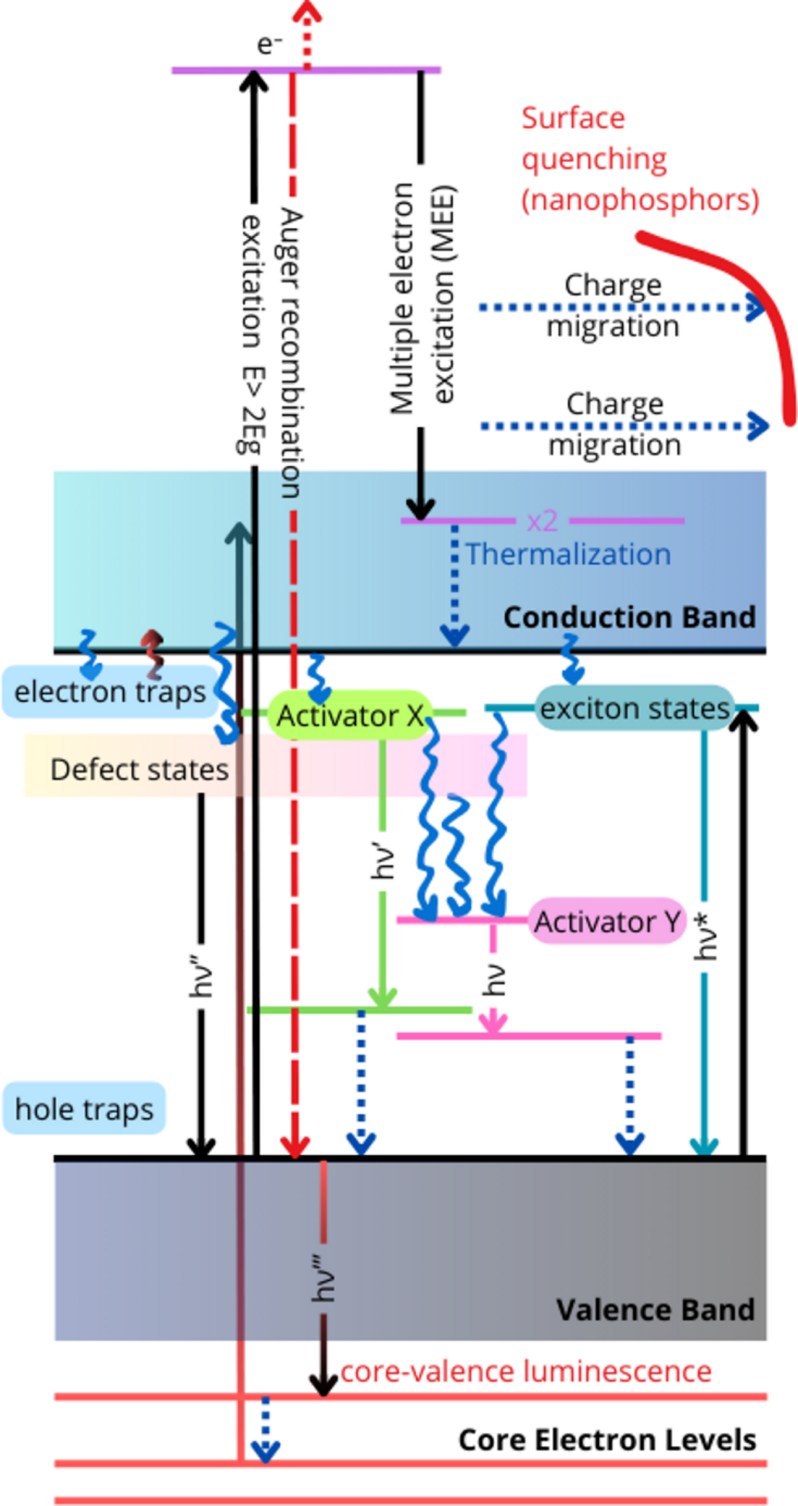
Simplified representation of the physical processes of the possible excitation, radiative *h*ν and non-radiative (wiggling arrows and dashed arrows) transitions in doped and pristine insulator materials, based on a literature review of the recent papers citing P66 (see Section 6 – *Scientific case* – and references therein).

A tentative scheme of the states and transitions involved in the processes occurring after absorbing the VUV-energy photons is presented in Fig. 7 ▸.

The excitation of a wide-bandgap material with energies higher than the bandgap result in the production of an electron in the conduction band. Multiple relaxation pathways are then possible for this excited state, depending on the material’s chemical composition and structural characteristics.

Radiative relaxation pathways can be categorized by their origin entity: the host, the dopant and the defects. Free and STE, donor–acceptor pair recombination, the emission from matrix ions, and cross-luminescence can be attributed to the first category. The defects, *e.g.* colour centres (electrons trapped by anion vacancies in halogen inorganic salts), antisite defects *etc.* can appear after exposure of the material to high-energy radiation or extreme conditions (Museur *et al.*, 2025 ▸). The emission spectra of the dopants are also sensitive to the presence of the emitting ion in structurally non-equivalent sites, *e.g.* Pr^3+^ in Pr(I) and Pr(II) sites in LiNaY_2_F_8_ (Zdeb-Stańczykowska *et al.*, 2024 ▸). Similarly, Ti^4+^ in Hf^4+^ or in Ge^4+^ sites in HfGeO_4_:Ti (Jedoń *et al.*, 2023 ▸), Ce^3+^ in Ce1 and Ce2 sites in garnets (Berezovskaya *et al.*, 2022 ▸; Shakhno *et al.*, 2024 ▸), or Cr^3+^ impurities on the surface under a weak crystal field (broad ^4^T_2_–^4^A_2_ emission) and in the bulk under strong crystal field (structured ^2^*E*–^4^*A*_2_ emission) (Spiridigliozzi *et al.*, 2023 ▸) exhibit different emission band positions and kinetic characteristics.

Nonradiative relaxation pathways are mostly related to the energy dissipation through the crystal lattice vibration (the phonon relaxation mechanism) and can be suppressed in experiments at low temperatures (*e.g.* 10 K), accessible at P66. Another type of nonradiative relaxation mechanism is the capture of the electron in the conduction band by the electron traps. In nanomaterials, a surface nonradiative relaxation after the charge migration has been reported by Pankratov *et al.* (2022 ▸).

The energy-transfer processes between the matrix and a dopant or between dopants can be revealed by comparing the time-integrated fingerprint emission and excitation spectra and studying the kinetic details of the emission components. Various energy transfer schemes, *e.g.* matrix–dopant (Demkiv *et al.*, 2024 ▸; Bartosiewicz *et al.*, 2024 ▸; Zhunusbekov *et al.*, 2024 ▸; Chukova *et al.*, 2024 ▸; Zdeb-Stańczykowska *et al.*, 2025 ▸), defect–dopant (Zdeb-Stańczykowska *et al.*, 2024 ▸), organic antenna–dopant (Teotonio *et al.*, 2025 ▸) and dopant–dopant in doubly-doped materials [*e.g.* Lu_2_SiO_5_:Ce,Yb (Viahin *et al.*, 2023 ▸)] can be used as a way to control the relaxation pathway of the excited state.

Understanding the potential mechanisms of excitation energy relaxation is essential for guiding the design and selection of promising scintillator materials.

### 6.2. The focus of fundamental and applied research at P66

The experiments carried out at the PETRA III P66 beamline are mainly focused on materials science. The applied research targets:

(1) Novel scintillator materials for imaging and radiation safety (Majewski-Napierkowski *et al.*, 2023 ▸; Jedoń *et al.*, 2023 ▸; Zaffalon *et al.*, 2023 ▸; Demkiv *et al.*, 2024 ▸; Pankratova *et al.*, 2024 ▸; Bartosiewicz *et al.*, 2024 ▸; Zdeb-Stańczykowska *et al.*, 2024 ▸), following the ever-increasing modern demands, *e.g.* fast sub-10 ps scintillators for time-of-flight positron emission tomography, with high light yield and low afterglow (Viahin *et al.*, 2023 ▸; Erroi *et al.*, 2023 ▸; Jarý *et al.*, 2023 ▸).

(2) Materials for lighting devices [*e.g.* VIS and UVC phosphors (Zdeb-Stańczykowska *et al.*, 2024 ▸; Chornii *et al.*, 2024 ▸; Chukova *et al.*, 2024 ▸; Zdeb-Stańczykowska *et al.*, 2025 ▸; Rebrova *et al.*, 2025 ▸; Zhunusbekov *et al.*, 2024 ▸)], bright and cost-effective LEDs with high colour rendering index and possibility of colour temperature tuning (Majewski-Napierkowski *et al.*, 2024 ▸; Shakhno *et al.*, 2024 ▸; Teotonio *et al.*, 2025 ▸), luminescent paints for road markers and safety signs (Berezovskaya *et al.*, 2022 ▸).

(3) Temperature sensors (Jedoń *et al.*, 2023 ▸; Zdeb-Stańczykowska *et al.*, 2025 ▸; Rebrova *et al.*, 2025 ▸).

(4) Materials exposed to extreme conditions, perspective for VUV optic components or nuclear fusion equipment (Museur *et al.*, 2024 ▸; Spiridigliozzi *et al.*, 2023 ▸; Museur *et al.*, 2025 ▸).

The fundamental research conducted in VUV spectroscopy accesses the details of the electronic and chemical structure of materials, *e.g.*

(*a*) dopant crystallographic sites (Zdeb-Stańczykowska *et al.*, 2024 ▸, Zdeb-Stańczykowska *et al.*, 2025 ▸; Shakhno *et al.*, 2024 ▸; Mosafer *et al.*, 2023 ▸);

(*b*) oxidation states of activator ions (Demkiv *et al.*, 2024 ▸; Bartosiewicz *et al.*, 2024 ▸);

(*c*) vacancy and antisite defects (Jarý *et al.*, 2023 ▸; Zhunusbekov *et al.*, 2024 ▸; Museur *et al.*, 2024 ▸);

(*d*) influence of additives on the vacancy defects, ruling the quantum efficiency of radiative relaxation of an exciton (Pidhornyi *et al.*, 2023 ▸);

(*e*) unit-cell volume changes upon doping (Pidhornyi *et al.*, 2023 ▸; Mosafer *et al.*, 2023 ▸).

Both applied and fundamental research projects, performed at the beamline, play a vital role in training Masters and PhD students. This enhances the socioeconomic impact of the beamline beyond the scientific results and industrial R&D applications, contributing to the shaping the next generation of academics.

### 6.3. Typical categories of materials under study using time-resolved VUV radiation

Along with wide-bandgap ‘bulk’ materials such as single crystals (Demkiv *et al.*, 2024 ▸; Pankratova *et al.*, 2024 ▸; Bartosiewicz *et al.*, 2024 ▸; Chornii *et al.*, 2024 ▸; Shakhno *et al.*, 2024 ▸; Museur *et al.*, 2024 ▸; Pidhornyi *et al.*, 2023 ▸), ceramics (Zhunusbekov *et al.*, 2024 ▸; Zhunusbekov *et al.*, 2025 ▸) and glass-ceramics (Chukova *et al.*, 2024 ▸), powders (Jedoń *et al.*, 2023 ▸; Zaffalon *et al.*, 2023 ▸; Zdeb-Stańczykowska *et al.*, 2024 ▸; Zdeb-Stańczykowska *et al.*, 2025 ▸; Rebrova *et al.*, 2025 ▸; Mosafer *et al.*, 2023 ▸), and single-crystalline films (Majewski-Napierkowski *et al.*, 2024 ▸), the luminescence properties of the nanoparticles have been shown to be rather distinct due to their large surface (Spiridigliozzi *et al.*, 2023 ▸; Chukova *et al.*, 2024 ▸). Indeed, the passivation of the surface of the nanoparticle was shown to possibly improve its characteristics: when incorporated in the polymethyl­methacrylate/polylaurethmethacrylate matrix, nanocrystalline lead halide perovskite particle (LH-NCs) optical properties were significantly improved comparatively to the bare LH-NCs (Erroi *et al.*, 2023 ▸): their emission spectrum becomes narrower, showing a single excitonic emission (contrarily to the three-band structure in bare NCs) and the luminescence quantum yield that increased almost twofold, reaching 90%. The kinetics measurements, using excitations of energies below and above the ionization potential of LH-NCs, show considerable acceleration, interpreted as a dominating influence of the nonradiative Auger recombination on the charge carrier dynamics under high-energy radiation. The nature and types of defects (Jarý *et al.*, 2023 ▸) and, on the other hand, assignment of luminescence bands to donor–acceptor recombination relaxation mechanisms (Rogulis *et al.*, 2022 ▸) is particularly efficiently addressed using a combination of electron paramagnetic resonance and VUV time-resolved luminescence.

The hybrid organic–inorganic materials (Erroi *et al.*, 2023 ▸; Chukova *et al.*, 2024 ▸; Teotonio *et al.*, 2025 ▸) and semiconductor quantum wells (QWs) (Hájek *et al.*, 2024 ▸) complement the variety of the studied structural architectures. The InGaN/GaN multiple QW luminescence was attributed to the InGaN QW grown on the *c* plane (2.98 eV) and to the QW grown on V-pit sidewalls (3.28 eV). The luminescence kinetics analysis of the first band suggested the importance of the non-radiative surface recombination, in line with the trend off between the absorption and excitation spectra in the range 6–6.9 eV. Multiple electron excitation, Auger recombination and ionization processes have been shown to contribute and bring structure in the excitation spectra and decay kinetics for the excitations well above the bandgap (Pankratov *et al.*, 2022 ▸; Erroi *et al.*, 2023 ▸; Jarý *et al.*, 2023 ▸; Hájek *et al.*, 2024 ▸; Popielarski *et al.*, 2023 ▸). In this direction, core electron excitation to the conduction band can also lead to the so-called cross-luminescence (a radiative transition of the valence electrons to the outermost core level) (Gundacker *et al.*, 2021 ▸).

## 7. Conclusions

The PETRA III P66 beamline for time-resolved luminescence spectroscopy (inheriting its concept from the SUPERLUMI beamline) has been constructed with the primary and the VUV Pouey monochromators refurbished and relocated from the parent station. It was open for the first users in late 2021 and achieved the desired experimental performance in 2024. The reconstructed beamline covers the 3.7–40 eV photon energy excitation range with the highest 4 × 10^12^ photons s^−1^ flux at the sample at 7.75 eV (160 nm). Luminescence emission can be registered in the 115–1200 nm spectral range with 130 ps time resolution. Transmission and specular reflection measurements are also possible. The sample environment at the P66 allows us to vary the temperature in the 10–700 K range under UHV conditions. Luminescence measurements are possible for all types of solid-state samples that satisfy the UHV conditions of the sample chamber. The scientific case of the PETRA III P66 beamline has also been discussed.

## Figures and Tables

**Table 1:** Specifications of the PETRA III P66 beamline

Source	Bending magnet				

Mirror	Focusing plane	Shape	Angle (°)	Material (surface/substrate)	Mirror area, *L* × *W* (mm)
M1	Horizontal	Plane-cylindric	15	Gold/mono-Si 〈100〉	100 × 160
M2	Vertical	Plane-elliptic	15	Gold/fused silica	200 × 60
M3	Horizontal/vertical	Toroid	12	Gold/fused silica	80 × 40

Ring current	100 mA (timing mode) / 120 mA (multi bunch mode)				
Tuneable excitation energy range	3.7–40 eV				
Time interval	192 ns (40 bunches) / 16 ns (480 bunches)				
Time resolution	130 ps, Instrument Response Function IRF of the MicroChannel Plate PhotoMultiplier Tube MCP-PMT				
Polarization	Horizontal				
Focal spot size on the sample	0.5 mm × 4 mm				
Adjustable PM slit width	200–2500 µm (corresponding to spectral resolution of 0.08–0.63 nm)				
Flux at sample	4 × 10^12^ photons s^−1^ at 7.75 eV (160 nm)				
Spectral range of the secondary monochromators	Andor Kymera 328i: 180–1200 nm				
	VUV Pouey: 50–300 nm				
Spectral range of the photodetectors	250–1200 nm (Newton 920 CCD camera from Andor)				
	180–830 nm (Hamamatsu R6358 PMT)				
	160–850 nm (Hamamatsu R3809U-50 MCP-PMT)				
	115–320 nm (Hamamatsu Solar Blind R6836 PMT)				
Sample temperature range	10–700 K				

## Data Availability

The data reported in the article are available upon request to the corresponding author.
